# Sustainable Strategies to Prevent Iron Deficiency, Improve Yield and Berry Composition in Blueberry (*Vaccinium* spp.)

**DOI:** 10.3389/fpls.2019.00255

**Published:** 2019-03-12

**Authors:** Lucía Michel, Álvaro Peña, Claudio Pastenes, Pablo Berríos, Adamo Domenico Rombolà, José Ignacio Covarrubias

**Affiliations:** ^1^Facultad de Ciencias Agronómicas, Universidad de Chile, Santiago, Chile; ^2^Department of Agricultural and Food Sciences, University of Bologna, Bologna, Italy

**Keywords:** intercropping, Fe-heme, iron deficiency, phenolic compounds, fruit quality

## Abstract

The aim of this investigation was to study the effect of sustainable strategies to correct iron deficiency in blueberries, based on Fe-heme applications or intercropping with graminaceous species, on yield, and berry quality variables. The experiment was conducted in a blueberry orchard established in a sub-alkaline soil. The association with grasses increased the crop load and yield (only *Festuca rubra*), and decreased the skin/flesh ratio. In addition, these treatments increased anthocyanins as well as some hydroxybenzoic acids, hydroxycinnamic acids, flavanols, and flavonol concentrations in skins with a similar effectiveness as Fe-EDDHA, whereas the Fe-heme applications did not influence such parameters. Moreover, data revealed that the association with both grasses decreased the firmness of the berries, whereas none of the treatments assessed changed the soluble solids, pH, acidity, and the soluble solids/acidity rate compared to the control. These results suggest that Fe nutrition is crucial for yield and berry quality in blueberry, and that intercropping with grasses may be an effective and sustainable alternative to counteract Fe deficiency in blueberry, with a similar effect on berries to that achieved with Fe-EDDHA.

## Introduction

Recently, the daily consumption of functional foods for the prevention of some human diseases has become highly promoted by several health organizations in the world. In this context, the demand for blueberries (*Vaccinium* spp.) has progressively increased in some countries due to the high phenolic compounds concentration in their skins, which are characterized by a great antioxidant capacity (Cordes et al., 2016). Blueberry is a species evolved in rainy areas characterized by soils with an acidic pH from 4.5 to 5.5 (Retamales and Hancock, 2012), and when it is cultivated in lower rainfall zones, often characterized by alkaline or sub-alkaline soils, frequently manifest severe symptoms of iron (Fe) deficiency, drastically reducing plant growth and yield (Rombolà and Tagliavini, 2006).

Even though a few studies have assessed the effect of Fe deficiency on fruit chemical composition and quality in some fruit crops, knowledge regarding the impact of Fe chlorosis on blueberry quality, and healthiness is scarce. In peach fruits, some experiences indicate that Fe deficiency decreases fruit firmness and the red skin color, increasing organic anion concentrations (particularly succinate and quinate), vitamin C, and total phenolic compounds (Álvarez-Fernández et al., 2003). In grapevines, Fe deficiency has been shown to increase the phenolic compounds accumulation in berries (Bavaresco et al., 2010). In contrast, in the model plant *Perilla frutescens*, anthocyanin synthesis was negatively affected by Fe deficiency, likely associated to a reduced activity of the anthocyanidin synthase (ANS), an enzyme involved in the anthocyanidin synthesis (Saito et al., 1999; Turnbull et al., 2000; Nakajima et al., 2001), and whose activity requires a ferrous Fe center. The controversy on the impact of Fe deficiency on the secondary metabolites concentration in fruits might be due to a subtle effect of the environment on the biosynthetic regulatory signals, or a species dependent sensitivity. Even though blueberries are considered as a healthy nutritional fresh produce, precisely because of their fruit chemical composition, and also as a highly sensitive crop to Fe deficiency, rather scarce knowledge is available in this regard.

In blueberry orchards located in sub-alkaline areas, soil acidification by means of elemental sulfur (S) previous to planting or with sulfuric or phosphoric acid applications during the growing season through the irrigation system, is a common field practice worldwide. In the former case, soil pH varies after long periods of time since microorganisms mediate the transformation of S into inorganic acids. Moreover, depending on the initial soil pH values and the buffer capacity of the soil, extremely high S amounts should be applied to shift the pH down to optimal levels for blueberry (Horneck et al., 2004). As for sulfuric acid applications, soil pH is more quickly reduced, especially in weakly buffered soils. However, this material is hazardous and difficult for farm workers to use (Horneck et al., 2004), and repeated soil applications of sulfuric acid may have a detrimental effect on the microbial biomass and mycorrhizal fungi populations, and on carbon dioxide emissions to the atmosphere. On the other hand, the treatment for Fe chlorosis with Fe chelates is costly and not environmental nor human friendly (Rombolà and Tagliavini, 2006) due to the high risk of metals and chelating agents leaching into the deep soil layers and into the water table (Rombolà and Tagliavini, 2006).

The above mentioned evidences of the negative impact of traditional Fe corrective measures, have led to the use of natural Fe sources to control Fe deficiency. In this context, animal blood-derived compounds, composed of a relatively high Fe content (20–30 g Fe kg^-1^) chelated by a heme group related to hemoglobin (López-Rayo et al., 2015), have been shown to be highly stable and capable of maintaining available Fe for plants in calcareous soils (Yunta et al., 2013). In controlled experiments conducted on grapevines cultivated in a calcareous soil, Fe-heme applications have increased the leaf chlorophyll concentration, plant growth, and potassium content in leaves (López-Rayo et al., 2015). On the other hand, some investigations have reported that intercropping systems with grasses are effective at preventing Fe chlorosis in co-cultivated crops (Cañasveras et al., 2014, Covarrubias et al., 2014, Granja and Covarrubias, 2018). The mechanism involved in improving Fe nutrition in associated plants is attributed to the ability of monocotyledoneous species to extrude mugineic acid family phytosiderophores (MAs), which are chelating compounds that form a soluble complex with Fe^3+^, increasing its availability also for the associated crops (Takagi, 1976; Ma et al., 2003; Xiong et al., 2013). For example, some authors have described that the main phytosiderophore secreted by *Festuca rubra* is 2′-deoxymugineic acid (DMA), whereas the graminaceous species *Poa pratensis* exudes DMA, avenic acid A (AVA) and 2′hydroxyavenic acid A (HAVA) (Ma et al., 2003; Ueno et al., 2007). Other studies have reported positive results from this sustainable management technique to prevent Fe chlorosis in fruit tree crops (Cañasveras et al., 2014, Covarrubias et al., 2014), however, to date there have been no reports published on a species highly susceptible to Fe deficiency like blueberry. In addition, some authors have described the possibility of the Fe^3+^-DMA complex being directly absorbed by intercropped peanut roots, since a functional Fe^3+^-DMA transporter, AhYSL1, was identified in this species (Xiong et al., 2013).

Even though investigations have been conducted on the effect of alternative and more sustainable management techniques for Fe correction in calcareous soils in blueberry productive and vegetative variables (Michel et al., 2019), the impact on the berry composition is lacking. Indeed, the use of animal blood-derived compounds and/or intercropping with graminaceous species implies the modification of soil nutrition properties related to nitrogen (N) availability, eventual phytoalexins, or simply modification of the competition for light, water and nutrients with eventual impacts on the fruit yield and metabolism (Michel et al., 2019). Accordingly, the present study is focused to evaluate the effect of Fe-heme applications or intercropping with graminaceous species on productive and berry quality variables in blueberry cv. Emerald, cultivated in a sub-alkaline soil.

## Materials and Methods

### Plant Material, Treatments, and Design of the Experiment

The experiment was undertaken from May 2014 to April 2015 (2014–2015 season) in a blueberry orchard located in the Valparaiso Region, Chile (32°42^′^S and 70°54^′^W) in an alluvial soil composed of 4% total calcium carbonate, 2% active lime, 1.1% organic matter and pH 8.1. These properties provided chemical conditions inducing Fe chlorosis in blueberries. The trial was conducted on highbush blueberries cv. Emerald (interspecific origin based largely on *Vaccinium corymbosum* L. with some genes from *Vaccinium darrowi* Camp), planted in September 2011 at a distance of 3 m between the rows and 0.8 m between plants along the row.

In autumn 2014, five contiguous rows were selected, homogeneous in size and degree of Fe chlorosis in the plants. The treatments tested were: (1) Control: bare soil; (2) Soil-applied Fe-ethylenediamine-N,N’-bis (2-hydroxyphenyl)acetic acid (Fe-EDDHA) chelate; (3) Soil-applied bovine blood compound (Fe-heme); (4) intercropping with *F. rubra* (graminaceous species), and (5) intercropping with *P. pratensis* L. (graminaceous species). A Latin square design (5 × 5) was used to take into account the slope and drip line as possible independent sources of variance. Each treatment was replicated five times. The experimental plot for each treatment was composed of 6 plants, and the treatments along the same row were separated by 2 plants between them.

### Experimental Conditions and Sample Collection

The Fe chelate was applied to the soil from August 2014 according to the SPAD value in order to maintain an intensive green color in the leaves (SPAD index > 35). Doses of 500 mL per plant of a 5% Fe-EDDHA solution (4 g Fe-EDDHA L^-1^) were occasionally applied to the soil, reaching 1 g of Fe applied per plant at the end of the season. The Fe-heme was applied through a dried bovine blood formulation composed of 2,675 mg Fe kg^-1^. Bovine blood was diluted in distilled water to a concentration of 20 g L^-1^ and applied to the soil at doses of 500 mL per plant every 15 days, reaching 0.27 g of Fe applied per plant at the end of the season. The Fe-EDDHA and Fe-heme applications were carried out manually, from the onset of bud break (spring 2014) to the summer flush growth. The graminaceous species *F. rubra* L. and *P. pratensis* L were sown over the rows in autumn 2014 at a density of 20,000 seeds m^-2^. During the season, the graminaceous species were cut manually to a height of 5 cm every time they reached 15 cm. The plants were irrigated daily through two 2 L h^-1^ in-line microdrip emitters, maintaining a constant soil moisture level, close to field capacity (40% saturation). The soil water content was measured daily using one tensiometer per experimental plot. In intercropped plants, an additional water supply was added according to the tensiometers record to maintain a similar soil moisture between treatments. The agronomic management of pruning, phytosanitary control, and fertilization with macro and micronutrients, except for Fe, were performed regularly throughout the season.

Leaf chlorophyll concentration is an accepted tool to monitor Fe status in fruit crops provided other nutrient deficiencies are excluded, given that leaf Fe concentrations cannot be used for this purpose (Römheld, 2000; Kosegarten et al., 2001). At harvest time, the leaf chlorophyll concentration (μg cm^-2^) was measured on the first completely expanded leaf of 16 shoots per experimental plot.

All harvested fruits were manually collected, when the skin color reached the 75% Blue stage, used for commercial purposes. The harvest took place on 5 different days, according to the fruit maturation rate. At each harvest, yield, and number of fruits per plant were measured for 6 plants in each replicate. At the end of the season, the total yield and crop load recorded at each harvest were determined, and the average berry weight was calculated.

In the third harvest (December 11, 2014), which was the largest and most representative (55% of the harvested fruit), berry samples (one composed of 40 and two of 200 berries) were collected from each experimental unit. The berries were sampled at random, considering that their size was representative of the experimental unit. The samples composed of 40 berries were used for firmness determinations, whereas one of the samples composed of 200 berries was used for chemical analysis, and the others were frozen at -80°C for phenolic compound determinations.

### Firmness of Berries

Firmness of the berries was measured in samples of 40 berries per experimental unit, within a few hours of the harvest. Firmness was determined in each berry using a fruit texture analyzer (FTA 65-14, GÜSS, Strand, South Africa) with a plunger 3 cm in diameter.

### Physio-Chemical Analysis of Berries

These measurements were taken in samples of 200 berries per experimental unit. Blueberries were peeled, weighed, and the skin/flesh ratio was determined. Successively, a composite sample was made by mixing and grinding the mesocarp of the 200 fruits per experimental unit. The ground pulp was then filtered and divided into two aliquots for chemical analysis. Soluble solids (SS), reported as °Brix, were determined with a digital refractometer thermocompensated at 20°C (PAL-1, Atago Co, Ltd., Tokyo, Japan). Titratable acidity (expressed as a percentage of citric acid) was measured with NaOH 0.1 N up to pH 8.2, and the pH of the mesocarp was measured with a pH meter (pHep-HI98107, Hanna Instrument, Padua, Spain).

### Determination of Phenolic Compounds

For the extraction, the skins of 200 berries per experimental unit, frozen at -80°C, were peeled, weighed and placed in a methanol:water solution (80:20 v/v) at a 1:5 ratio. The samples were homogenized, shaken (BioScan OS-20, LabTec, Chile) for 30 min and centrifuged (Universal 320, Hettich Lab, Germany) for 10 min at 1,500 rpm at 20°C. Successively, the liquid part was refrigerated and the supernatant was again macerated, shaken and centrifuged, following the previous procedure. Once this second extraction was complete, the liquid fraction was recovered and added to the first, and the final solution was centrifuged at 4,000 rpm at 20°C for 15 min. Thereafter, the samples were filtered under vacuum through a PVDF membrane, porosity 8 μm.

Total anthocyanin concentration was determined according to García-Barceló (1990), based on bisulfite discoloration. The extract was diluted 100 times, and 1 mL of the diluted extract was added with 1 mL of acid alcohol and 20 mL of 2% HCl. The resulting solution was separated into two test tubes, and to each one 4 mL of NaHSO_3_ (sodium metabisulfite), and 4 mL of distilled water were added. After placing the tubes in the dark for 20 min, absorbance was measured by spectrophotometry (Lambda 25, PerkinElmer, Hartford, United States) at 520 nm with 10 mm glass cuvettes. Total phenols concentration was determined in the extract (diluted at 1:100) according to García-Barceló (1990), using gallic acid as the standard. The analysis was performed by spectrophotometry at 280 nm with 10 mm quartz cuvettes. Total tannins concentrations were assessed as described by the Bate-Smith (1972) methodology, which is based on tannins hydrolysis in acidic medium. After 1:50 dilution of the extract, 4 mL of the sample were placed in two test tubes, and 2 mL of distilled water and 6 mL of 35% HCl were added. Then, one tube was subjected to a water bath at 90°C for 30 min, and the other at room temperature. Both samples were evaluated with spectrophotometry at 550 nm with 10 mm glass cuvettes.

The anthocyanins profile were determined according to Peña-Neira et al. (2007), using high-performance liquid chromatography (Agilent Technologies 1100 series HPLC system, Santa Clara, CA, United States), consisting of a photodiode array detector (DAD), model G1315B; a quaternary pump, model QuatPump G1311A; a degasser, model G1379A; and an autosampler, model G1329A. A RP-18 reverse-phase column (Merck Hitachi, Tokyo, Japan), 5 μm pore size, 250 mm × 4 mm was used. One-hundred mL of each extract was filtered through a 0.45 mm pore size and then subjected to reverse-phase chromatographic separation at 20°C. The quantification was carried out by peak area measurements at 520 nm. Anthocyanins were quantified and expressed as mg of g^-1^ of berry skin of malvidin-3-glucoside. The calibration curves at 520 nm were obtained by injecting different volumes of standard solutions under the same conditions as for the samples analyzed.

The low molecular weight phenolic compounds were quantified as described by Peña-Neira et al. (2007), using the same Agilent model HPLC-DAD equipment as described for anthocyanin determinations. A Nova Pack C18 reverse-phase column (4 μm, 3.9 mm i.d. × 300 mm; Waters Corp.) was used. Both chromatographs were coupled to an Agilent Chem Station (version B.04.03) data processing station. Signals were detected at 280 nm, at 20°C and with an injection volume of 10 μm. The low molecular weight phenolic compounds were identified by comparing their absorption spectrum and retention time to their respective standard. The calibration curves at 280 nm were obtained by injecting different volumes of standard solutions under the same conditions as for the samples analyzed. The phenolic compounds were expressed as mg of each phenolic compound g^-1^ of berry skin.

### Statistics

The analysis of variance (ANOVA) was performed under the framework of mixed linear models (MLM). In the case of significant differences between treatments, the DGC test for multiple comparisons was used (α = 0.05). The statistical software used was InfoStat v. 2013.

## Results

### Leaf Chlorophyll Concentration, Crop Load, Plant Production, Berry Weight, and Skin/Flesh Ratio

At harvest time, the leaf chlorophyll concentration (μg cm^-2^) average measured on the first completely expanded leaf of 16 shoots per experimental plot was the following: (1) Control: 7.3 units ± 1.6; (2) Soil-applied Fe-EDDHA chelate: 13.1units ± 1.5; (3) Soil-applied Fe-heme: 8.1 units ± 1.7; (4) Intercropping with *P. pratensis* L.: 9.8 units ± 1.6; (5) Intercropping with *F. rubra*: 11.6 units ± 1.6. Such leaf chlorosis is attributed to Fe deficiency since the leaf mineral concentration analysis revealed values in the sufficiency range for the other nutrients (data not reported). In addition, plants treated with Fe-EDDHA showed the highest values, with significant differences in comparison with the control plants.

**FIGURE 1:**
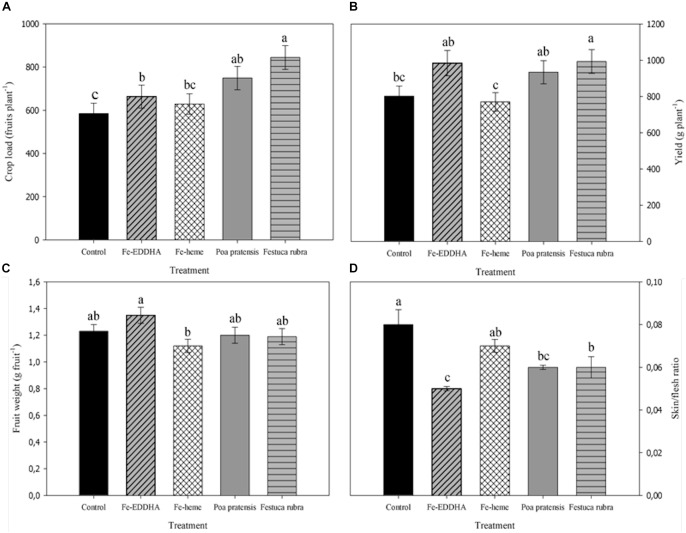
Crop load **(A)**, yield **(B)**, berry weight **(C)**, and skin/flesh ratio **(D)** at harvest in “Emerald” blueberries under different treatments to prevent Fe deficiency. Adjusted means with different letters between treatments indicate significant differences according to DGC test (*p* < 0.05). Vertical bars indicate the standard error.

The association with *F. rubra* and *P. pratensis* as well as the Fe-EDDHA applications increased the crop load, whereas the fertilization with Fe-heme did not modify this variable compared to control plants (Figure 1A). As for yield, however, only the intercropping with *F. rubra* treatment increased this variable compared to control, while the Fe-heme treated plants were the less yielding plants, similar to untreated plants (Figure 1B). Regarding the berry weight, in general no marked differences were observed between treatments, but two opposite and significant results were clear between the Fe-EDDHA and the Fe-heme treated plants, the latter with the lowest berry weight (Figure 1C). Also, regarding fruit physical properties, some treatments reduced the skin to flesh weight ratio, such as the intercropping with *F. rubra* and *P. pratensis* and, particularly, fruits from plants treated with the Fe-EDDHA chelate, all of them with significant lower averages of this parameter compared to control fruits (Figure 1D).

### Firmness and Chemical Berry Composition

From Table 1, it is clear that none of the Fe correction treatments induced significant changes on the ripening of fruits since the SS concentration, pH, acidity and the SS/acidity ratio were similar between treatments. In fact, the extreme values for each of these parameters between treatments accounted for an 11, 3, 20, and 13% for SS, pH, acidity and SS to acidity ratio, respectively. As for fruit firmness, however, differences were observed between treatments, in which the association with both grasses decreased the firmness compared with the control and with plants treated with Fe-heme, whereas the berries collected from plants treated with Fe-EDDHA reached intermediate values (Table 1).

**Table 1:** Firmness (N), soluble solids (°Brix), pH, acidity (%), and soluble solids/acidity ratio at harvest in “Emerald” blueberries under different treatments to prevent Fe deficiency.

Treatment	Firmness	Soluble solids	pH	Acidity	Soluble
	(N)	(°Brix)		(% citric acid)	solids/acidity
Control	3.11 ± 0.07 a	15.9 ± 0.19	3.63 ± 0.06	0.45 ± 0.03	33.1 ± 4.03
Fe-EDDHA	2.99 ± 0.07 ab	16.5 ± 1.23	3.68 ± 0.06	0.58 ± 0.04	28.7 ± 1.04
Fe-heme	3.07 ± 0.07 a	14.6 ± 0.59	3.69 ± 0.05	0.48 ± 0.03	30.5 ± 1.82
*Poa pratensis*	2.91 ± 0.07 bc	15.1 ± 0.58	3.57 ± 0.07	0.50 ± 0.02	32.6 ± 3.44
*Festuca rubra*	2.88 ± 0.07 c	14.9 ± 0.63	3.62 ± 0.06	0.56 ± 0.03	28.9 ± 1.11
Significance	*p* = 0.0016	NS	NS	NS	NS


*In each column the adjusted mean ± standard error is presented. Adjusted means with different letters in the same column indicate significant differences between treatments according to the DGC test (*p* < 0.05). NS, not significant.*

### Phenolic Compounds Concentration in Berry Skins

The Fe treatments, except for the Fe-heme, had an impact on the berry skin composition of the blueberries considering total anthocyanins and total phenols, but no effect was observed in total tannins (Table 2). Regarding total anthocyanins, the intercropping and the Fe-EDDHA applications were the more promising treatments. As for total phenols, the intercropping treatments and the Fe-EDDHA applications were equally effective, inducing nearly a 40% increase in the concentration, in average, compared to control (Table 2).

**Table 2:** Total anthocyanins (mg g skin^-1^), total phenols (mg g skin^-1^) and total tannins (mg g skin^-1^) at harvest in “Emerald” blueberries under different treatments to prevent Fe deficiency.

Treatment	Total anthocyanins	Total phenols	Total tannins
	(mg g skin^-1^)	(mg g skin^-1^)	(mg g skin^-1^)
Control	13.7 ± 1.6c	54.3 ± 5.5b	0.38 ± 0.03
Fe-EDDHA	24.2 ± 1.6a	78.1 ± 5.4a	0.43 ± 0.01
Fe-heme	14.7 ± 1.8c	53.2 ± 5.5b	0.41 ± 0.02
*Poa pratensis*	21.8 ± 1.8b	75.0 ± 5.3a	0.40 ± 0.03
*Festuca rubra*	19.5 ± 2.1b	72.4 ± 4.8a	0.36 ± 0.02
Significance	*p* < 0.0001	*p* = 0.0193	NS


*In each column the adjusted mean ± standard error is presented. Adjusted means with different letters in the same column indicate significant differences between treatments according to the DGC test (*p* < 0.05). NS, not significant.*

Interestingly, the treatments implemented in the present study not only affected the total anthocyanins in the blueberry skins, but also its composition. Regardless of the treatment, four anthocyanin classes were found; all of them were 3-O-glycoside conjugates: malvidin, delphinidin, cyanidin and petunidin (Table 3). Glycosylation with galactose, glucose and arabinose were the forms found for malvidin and delphnidin, only galactose for cyanidin and, for petunidin, galactose, and arabinose additions were detected (Table 3). Of all, malvidin 3-galactoside and delphinidin 3-galactoside were the most abundant forms found in the berry skins. In agreement with data from Table 2, the higher concentrations of anthocyanins corresponded to berries from plants treated with Fe-EDDHA and from those intercropped with grasses (Table 3). In general, the Fe-EDDHA applications and the association with *P. pratensis* increased the malvidin, delphinidin and petunidin glycosides and cyanidin-3-galactoside concentrations in comparison with the control and Fe-heme plants (Table 3), whereas the association with *F. rubra* increased only delphinidin and some malvidin glycosides (Table 3). The Fe-heme applications, on the other hand, did not influence the skin anthocyanin composition compared to the control (Table 3).

**Table 3:** Anthocyanin (mg g skin^-1^) profile at harvest in ‘Emerald’ blueberries under different treatments to prevent Fe deficiency.

Anthocyanins (mg g skin^-1^)	Treatment					
	Control	Fe-EDDHA	Fe-heme	*Poa pratensis*	*Festuca rubra*	Significance
Malvidin 3-galactoside	0.080 ± 0.010bc	0.110 ± 0.010a	0.070 ± 0.010c	0.100 ± 0.010ab	0.100 ± 0.010ab	*p* = 0.0180
Malvidin 3-glucoside	0.039 ± 0.004c	0.062 ± 0.004a	0.037 ± 0.004bc	0.057 ± 0.004a	0.052 ± 0.004ab	*p* = 0.0053
Malvidin 3-arabinoside	0.030 ± 0.003b	0.050 ± 0.003a	0.030 ± 0.003b	0.050 ± 0.003a	0.050 ± 0.003a	*p* = 0.0134
Delphinidin 3-galactoside	0.080 ± 0.010c	0.120 ± 0.010a	0.090 ± 0.010bc	0.120 ± 0.010a	0.110 ± 0.010ab	*p* = 0.0084
Delphinidin 3-glucoside	0.026 ± 0.007b	0.039 ± 0.007a	0.026 ± 0.007b	0.037 ± 0.007a	0.034 ± 0.007a	*p* = 0.0063
Delphinidin 3-arabinoside	0.050 ± 0.010b	0.070 ± 0.004a	0.050 ± 0.004b	0.070 ± 0.010a	0.070 ± 0.010a	*p* = 0.0104
Cyanidin 3-galactoside	0.060 ± 0.010b	0.080 ± 0.004a	0.060 ± 0.004b	0.080 ± 0.010a	0.070 ± 0.010ab	*p* = 0.0185
Petunidin 3-galactoside	0.030 ± 0.002c	0.046 ± 0.002a	0.030 ± 0.002c	0.038 ± 0.002b	0.034 ± 0.002bc	*p* = 0.0011
Petunidin 3-arabinoside	0.026 ± 0.002b	0.036 ± 0.002a	0.025 ± 0.002b	0.034 ± 0.002a	0.031 ± 0.002ab	*p* = 0.0195


*In each column the adjusted mean ± standard error is presented. Adjusted means with different letters in the same column indicate significant differences between treatments according to the DGC test (*p* < 0.05).*

As for the phenolic acid compounds, the gallic and vanillic hydroxybenzoic acids and the chlorogenic and *trans*-caffeic hydroxycinnamic acids, were the most abundant on a weight basis in blueberry skins. Protocatechuic and ferulic acids were also detected but in a lower concentration and with no significant differences between treatments. Regarding hydroxybenzoic acids, the association with both grasses increased the gallic acid concentration as compared with the control berries, but not the Fe-heme treatment (Table 4). Also, the Fe-EDDHA applications increased the gallic and vanillinic acid berry skin concentrations compared to control (Table 4).

**Table 4:** Hydroxybenzoic acid (mg g skin^-1^) and hydroxycinnamic acid (mg g skin^-1^) profiles at harvest in “Emerald” blueberries under different treatments to prevent Fe deficiency.

Treatment	Hydroxybenzoic acids			Hydroxycinnamic acids		
	(mg g skin^-1^)			(mg g skin^-1^)		
	Protocatechuic	Gallic	Vanillinic	Ferulic	Chlorogenic	*Trans* caffeic
Control	0.0043 ± 0.0007	0.025 ± 0.002c	0.046 ± 0.002b	0.006 ± 0.002	0.37 ± 0.08b	0.06 ± 0.01 b
Fe-EDDHA	0.0056 ± 0.0005	0.032 ± 0.001a	0.059 ± 0.002a	0.008 ± 0.002	0.70 ± 0.07a	0.10 ± 0.02 a
Fe-heme	0.0040 ± 0.0006	0.026 ± 0.002bc	0.043 ± 0.003b	0.006 ± 0.002	0.35 ± 0.07b	0.07 ± 0.01 b
*Poa pratensis*	0.0052 ± 0.0006	0.031 ± 0.003a	0.050 ± 0.002ab	0.009 ± 0.003	0.67 ± 0.07a	0.10 ± 0.01 a
*Festuca rubra*	0.0052 ± 0.0005	0.030 ± 0.002ab	0.049 ± 0.002ab	0.009 ± 0.004	0.73 ± 0.08a	0.09 ± 0.02 ab
Significance	NS	*p* = 0.0194	*p* = 0.029	NS	*p* < 0.0001	*p* = 0.0446


*In each column the adjusted mean ± standard error is presented. Adjusted means with different letters in the same column indicate significant differences between treatments, according to DGC test (*p* < 0.05). NS, not significant.*

As for hydroxycinnamic acids, the association with graminaceous species and the fertilization with Fe-EDDHA increased the chlorogenic acid concentration compared to both, control and the Fe-heme treatments (Table 4). Besides, the intercropping with *P. pratensis* and application of Fe-EDDHA increased the *trans*-caffeic acid in skins more than in the control and Fe-heme, whereas the association with *F. rubra* reached intermediate values (Table 4). The treatments did not modify the ferulic acid concentration in the berry skins (Table 4).

Results concerning flavanols in skins revealed that intercropping with graminaceous species and Fe-EDDHA treatments increased the catechin and epicatechin concentrations compared to the control, whereas Fe-heme did not influence such concentrations (Table 5). In the case of procyanidin dimmers, no differences were registered between treatments (Table 5). In the di-Oh flavonol, a trend similar to the catechin and epicatechin was registered for the astilbin acid concentration, whereas no differences were recorded for the flavonol quercetin. In the case of coumarins, data revealed that Fe-EDDHA increased the esculetin concentration in comparison with Fe-heme and the control, whereas intercropping treatments reached intermediate values (Table 5).

**Table 5:** Flavanols (mg g skin^-1^), flavonols (mg g skin^-1^) and coumarins (mg g skin^-1^) profiles at harvest in “Emerald” blueberries under different treatments to prevent Fe deficiency.

Treatment	Flavanols			Flavonols		Coumarins
	(mg g skin^-1^)			(mg g skin^-1^)		(mg g skin^-1^)
	Procyanidin	Catechin	Epicatechin	Quercetin	Astilbin	Esculetin
	dimmers					
Control	0.010 ± 0.003	0.04 ± 0.01d	0.10 ± 0.01c	0.016 ± 0.003	0.05 ± 0.02b	8.2E-05 ± 0.000 bc
Fe-EDDHA	0.014 ± 0.005	0.07 ± 0.02c	0.16 ± 0.01a	0.023 ± 0.003	0.14 ± 0.03a	1.2E-04 ± 0.000 a
Fe-heme	0.011 ± 0.003	0.05 ± 0.01d	0.11 ± 0.02bc	0.020 ± 0.002	0.07 ± 0.02b	6.3E-05 ± 0.000 c
*Poa pratensis*	0.011 ± 0.003	0.09 ± 0.01b	0.16 ± 0.01a	0.023 ± 0.004	0.17 ± 0.04a	9.7E-05 ± 0.000 ab
*Festuca rubra*	0.020 ± 0.006	0.13 ± 0.02a	0.14 ± 0.01b	0.019 ± 0.003	0.19 ± 0.04a	1.0E-04 ± 0.000 ab
Significance	NS	*p* < 0.0001	*p* < 0.0001	NS	*p* = 0.0007	*p* = 0.0016


*In each column the adjusted mean ± standard error is presented. Adjusted means with different letters in the same column indicate significant differences between treatments according to the DGC test (*p* < 0.05). NS, not significant.*

## Discussion

Data collected in our experiment indicate that those treatments resulting in higher leaf chlorophyll concentrations at harvest (see section “Materials and Methods”), such as intercropping with *F. rubra* and *P. pratensis*, together with Fe-EDDHA applications, were effective in inducing changes in some productive and berry morphological characteristics compared to the control and Fe-heme plants. Indeed, these treatments increased the fruit load and, for plants intercropped with *F. rubra*, also an increase in the yield per plant was evident. In addition, the same treatments decreased the skin/flesh ratio (Figure 1). The skin to flesh ratio is affected by the berry volume, as it might be the case for the Fe-EDDHA treated plants, with the highest fruit average weight as well as the lowest skin/flesh relationship (Figure 1C,D), but may be also due to a more thick epidermis tissue. The lower crop load recorded in plants with a lower leaf chlorophyll concentration, that is control and Fe-heme, could be related to a lower fruit set or a higher early abscission of berries in these plants, considering that Fe chlorosis promotes the expression of genes involved in the synthesis and signaling of ethylene, resulting in the abscission of young fruits (Iqbal et al., 2013). Also, blueberries are regarded as a highly sensitive species to sink/source relationship (Jorquera-Fontena et al., 2018; Petridis et al., 2018), where the non-structural carbon reserves status in shoots and storage organs are determinant in the extent of the post-fruit set fall (Buwalda and Smith, 1990; Mehouachi et al., 1995). In fact, it is well known that the Fe status of plants is closely linked to the photosynthetic capacity of leaves (Briat et al., 2007), and in citrus, Fe deficiency induced reductions in the carbon status of plants, increasing the early fruit fall (Gómez-Cadenas et al., 2000). In our study, a lower leaf net photosynthesis during the season was registered, precisely, in control and Fe-heme plants in comparison with those treated with Fe-EDDHA and intercropping with grasses (Michel et al., 2019). Likewise, the effect of Fe nutrition on the carbon availability within the plant could contribute to explain the higher pulp fraction in relation to the skin in berries collected from plants with a better Fe status, such as those treated with Fe-EDDHA and intercropped with graminaceous, as well as the higher yield in plants associated with *F. rubra* than the control plants.

The ripening process, on the other hand, regarding SS, pH, acidity and SS acidity ratio were not altered by any of the Fe-correcting strategies (Table 1). This result, suggests that any eventual impact of the treatments on yield was rather the result of a sink to source balance, with no impact on the primary metabolism of the berries. Similar results from Fe nutrition studies have been reported for peach, citrus and pear (Álvarez-Fernández et al., 2003, 2011; Bañuls et al., 2003). However, as for berry firmness, a relevant trait for post-harvest market considerations, differences between the treatments assessed in the present study were recorded (Table 1). Firmness is associated to a combination of ripening stages, water content and cell wall properties in berries. In blueberry, the berry firmness varies according the ripeness status, being maximum before berry veraison and progressively decreasing after it, and the SS to acidity ratio has been reported as a very reliable indicator of the ripening stage and fruit firmness (Moggia et al., 2017). As for our results, no significant differences in the SS/acidity ratio was observed between treatments, even though differences of up to a 13% between the minimum -Fe-EDDHA- and maximum –intercropping with *P. pratensis*- were detected, both treatments with significant differences in the berry firmness. This might suggest that the primary metabolites and their ratio, suggested as a proxy for ripening state in fruits, are highly variable as compared to firmness. Alternatively, a more subtle effect of the treatments might be indirectly affecting the water status and physical skin properties of the berries, such as plant vigor and light interception and temperature on fruits, but those were not evident from our observations.

Data concerning anthocyanin content in ‘Emerald’ blueberries from our study, revealed the presence of 9 different compounds in the skins (Table 3), while 13 were reported in Italy (Giovanelli and Buratti, 2009), 15 in France (Kader et al., 1996), 25 in Canada (Gao and Mazza, 1994), 15 in United States (Ballington et al., 1987; Mazza and Miniati, 1993), and 10 in other countries and other varieties (Sapers et al., 1984). The anthocyanin compounds identified in berry skins were galactosides, glucosides and arabinosides of delphinidin, cyanidin, petunidin and malvidin, similar to previous studies (Kader et al., 1996; Taruscio et al., 2004; Giovanelli and Buratti, 2009). However, and in contrast with other investigations, peonidin was not detected (Kader et al., 1996; Tomás-Barberán and Espín, 2001; Lohachoompol et al., 2008; Giovanelli and Buratti, 2009). In addition, the major anthocyanins concentration corresponded to delphinidin 3-galactoside and malvidin 3-galactoside, followed by cyanidin 3-galactoside and delphinidin 3-arabinoside. Also, and consistent with determinations from different *Vaccinium corymbosum* varieties, anthocyanin compounds reached 30–35% of total phenols (Moyer et al., 2002; Sellapan et al., 2002; Lee et al., 2004; Giovanelli and Buratti, 2009).

As for low molecular weight phenols, the hydroxycinnamic acids were the largest group contained in skins. Within that group, the highly antioxidant molecule chlorogenic acid predominated, while ferulic acid was present in lower concentrations (Table 4), similar to previous reports (Häkkinen and Törrönen, 2000; Taruscio et al., 2004). As for the hydroxybenzoic acids group, the highest compound identified was vanillinic acid, followed by gallic and protocatechuic acids (Table 4). In the flavanols group, epicatechin predominated over catechin (Table 5), as seen in nine different *Vaccinium* species (Taruscio et al., 2004). Between di-OH flavanols and flavonols, astilbin showed a higher concentration than quercetin, and no myricetin or kaempferol was detected (Table 5), as reported by Taruscio et al. (2004). In this sense, Kader et al. (1996) and Sellapan et al. (2002) found kaempferol, although in a smaller amount than quercetin, a molecule regarded as the more abundant flavonol in blueberry (Kader et al., 1996, Häkkinen et al., 1998). Up to our knowledge, this is the first report on the phenolic composition in “Emerald” blueberries grown in Chile. Thus, although the phenolic composition and antioxidant capacity of blueberry is more influenced by the genotype than by the growing season, it is advisable to evaluate the same species for several producing seasons in order to precisely determine whether the genotype maintains its phenolic composition over environmental variations.

It has been widely reported that changes in berry weight usually leads to changes in phenol concentration in berries, as these compounds are affected by the berry size due to a dilution factor (Ojeda et al., 2002; Roby et al., 2004). In contrast, our results did not reveal significant differences between treatments in total phenol, total anthocyanin and total tannin concentrations, when are expressed as mg g^-1^ berry (data not reported), despite the effect of treatments on the skin/flesh ratio. This suggest, that the phenol content in berries were not necessarily the result of berry size variations but to an actual positive differential synthesis vs. degradation activities in skins. Indeed, our results are expressed as for skin weight basis (Tables 2–5). Data related to total phenols and total anthocyanins (Table 2) as well as the anthocyanins and low molecular weight phenols profiles (Tables 3–5) revealed a higher accumulation of these compounds in skins from plants treated with Fe-EDDHA and in intercropped plants than the control and Fe-heme. These results suggest that by better counteracting the Fe deficiency of blueberry plants, the Fe-chelate as well as the intercropping treatments favored the berry fruits chemical composition. Fe is a cofactor of the ANS enzyme, which is involved in the anthocyanin synthesis pathway (Saito et al., 1999; Turnbull et al., 2000), the activity of which requires a ferrous Fe center to catalyze the reaction that converts dihydroflavones (flavan-3,4-diol) into anthocyanidins (Turnbull et al., 2004). Similarly, for the low molecular weight phenols, Fe is also involved in its synthesis pathway as a cofactor of flavanone-3β-hydroxylase (F3H) and flavonol synthase (FLS) enzymes, which catalyze the consecutive conversion of flavanones to flavanonols, and of flavanonols to flavonols, respectively. In this sense, the oxidation reactions involving Fe-dependent enzymes play a crucial role in flavonoid biosynthesis (Saito and Yamazaki, 2002; Springob et al., 2003), and clearly, corrective and effective field practices regarding Fe nutrition have a positive impact on berry quality in field grown blueberries.

On the other hand, data related to both cinnamic and benzoic acids suggest that Fe might also be participating in the non-flavonoid compounds concentration in blueberry skins, but further studies are needed to clarify this issue. The synthesis and accumulation of phenolic compounds are strongly influenced by the photosynthetic capacity of plants (Smart and Robinson, 1991) which, in our study, was higher in plants treated with Fe-EDDHA and intercropped with the graminaceous species (data not shown). In fact, the synthesis of the shikimate pathway precursors come from hexoses derived from photosynthesis, ending in the formation of polyphenols such as flavonols, flavanols, anthocyanins, or tannins. Whether the higher benzoic and cinnamic acid concentrations results purely from a more favorable carbon supply condition in the Fe chelate and intercropping with grasses treatments, or a more subtle effect are involved, as it could be the need for antioxidant defenses and related systemic acquired resistance in Fe-deficient plants, depleting benzoic acids intermediates, is not clear and needs further investigations.

In view of the strong and positive relationship among the antioxidant capacity and the total phenol and anthocyanin content (Ehlenfeldt and Prior, 2001; Moyer et al., 2002; Sellapan et al., 2002), the study of sustainable management techniques, such as intercropping with grasses, that affect the crop and influence the phenolic accumulation of the fruits, will be useful to providing the consumer with a better product.

## Data Availability

The datasets generated for this study are available on request to the corresponding author.

## Author Contributions

JC conceived the work and wrote the manuscript. LM conducted the experiment and measured the field. ÁP performed the chemical analysis in berries. CP, PB, and AR contributed to the manuscript writing.

## Conflict of Interest Statement

The authors declare that the research was conducted in the absence of any commercial or financial relationships that could be construed as a potential conflict of interest.
